# Epstein-Barr virus latent membrane protein-1 (LMP-1) 30-bp deletion and *Xho *I-loss is associated with type III nasopharyngeal carcinoma in Malaysia

**DOI:** 10.1186/1477-7819-6-18

**Published:** 2008-02-15

**Authors:** Hui Shien See, Yoke Yeow Yap, Wai Kien Yip, Heng Fong Seow

**Affiliations:** 1 Department of Pathology, Faculty of Medicine and Health Sciences, Universiti Putra Malaysia, Serdang, Malaysia; 2 Department of Surgery, Faculty of Medicine and Health Sciences, Universiti Putra Malaysia, Serdang, Malaysia

## Abstract

**Background:**

Nasopharyngeal carcinoma (NPC) is a human epithelial tumour with high prevalence amongst Chinese in Southern China and South East Asia and is associated with the Epstein-Barr virus (EBV). The viral genome harbours an oncogene, namely, the latent membrane protein 1 (LMP1) gene and known variants such as the 30-bp deletion and loss of *Xho*I restriction site have been found. Less is known about the relationship between these variants and the population characteristics and histological type.

**Methods:**

In this study, the EBV LMP1 gene variants from 42 NPC and 10 non-malignant archived formalin fixed, paraffin-embedded tissues, as well as plasma from another 35 patients with nasopharyngeal carcinoma were determined by using Polymerase Chain Reaction (PCR). Statistical analysis was performed by using SPSS programme.

**Results:**

LMP1 30-bp deletion was detected in 19/34 (55.9%) of NPC tissues, 7/29 (24.1%) of plasma but absent in non-malignant tissues (8/8). Coexistence of variants with and without 30bp deletion was found only in 5/29 (17.2%) plasma samples but not in NPC tissues. The loss of *Xho*I restriction site in LMP1 gene was found in 34/39 (87.2%) of the NPC tissues and 11/30 (36.7%) of plasma samples. None of the non-malignant nasopharyngeal tissues (8/8) harbour *Xho*I-loss variants. LMP1 30-bp deletion was detected in 16/18 Chinese versus 3/15 Malays and 13/16 type III (undifferentiated carcinoma) versus 1/6 type I (keratinizing squamous cell carcinoma). *Xho*I-loss was found in 19/19 Chinese versus 14/19 Malays and 18/18 type III (undifferentiated) versus 2/5 type I (keratinizing squamous cell carcinoma). Statistical analysis showed that these variants were associated with ethnic race (30-bp deletion, *p *< 0.05; *Xho*I-loss, *p *= 0.046) and histological type of NPC (30-bp deletion, *p *= 0.011; *Xho*I-loss, *p *= 0.006). Nineteen out of 32 NPC tissues (19/32; 59.4%) and 6/24 (25%) of plasma samples showed the coexistence of both the 30-bp deletion and the loss of *Xho*I restriction site. A significant relationship was found with the Chinese race but not histological type.

**Conclusion:**

The incidence rate of 56% for LMP1 30-bp deletion was lower compared to previously reported rates of 75–100% in NPC tissues. Coexistence of variants with and without 30-bp deletion was found only in 5/29 plasma samples. The incidence rate of *Xho*I restriction site loss in NPC was comparable to other studies from endemic regions such as Southern China. For the first time, the presence of LMP1 30-bp deletion or *Xho*I-loss was associated with the Chinese race and type III NPC. Both these variants were not found in non-malignant tissues. The influence of these variants on disease progression and outcome in Chinese and type III NPC requires further investigation.

## Background

Nasopharyngeal carcinoma (NPC) is a tumour arising from epithelial cells of the nasopharynx. The neoplasm is uncommon in most countries with age-adjusted incidence for both sexes of less than one per 100,000 populations [1]. However, NPC is endemic in southern China where it is the third most common form of malignancy amongst men, with incidence rates of between 15 and 50 per 100,000 [2]. In Malaysia, it is also the second most common cancer among males in Malaysia which constitutes 8.8% of total male cancers [3] with incidence rates of 18.1 and 7.4 per 100,000 populations for Chinese males and females, respectively. Lower rates of 7%, 1.5% and 2.6% were reported for Malay males, Malay females and Indian males, respectively. About 81% of the cases diagnosed were at advanced stage of the disease [4].

A unique feature of NPC is its strong association with Epstein-Barr virus (EBV). EBV DNA is consistently detected in patients with almost all nasopharyngeal cancers from regions of high and low incidence. EBV has been found to be present in all the NPC samples by various techniques such as PCR, *in situ *hybridization and immunohistochemistry staining [5]. Latent EBV infection has been shown to be an early event in the development of the cancer [6]. Among the latent gene products encoded by EBV, latent membrane protein 1 (LMP1) is particularly interesting because it displays classic oncogenic ability in rodent fibroblast transformation [7,8] and it is capable of inducing a range of phenotypic changes in both B cells and epithelial cells [9]. The importance of LMP1 in tumorigenesis of NPC *in vivo *is supported by the finding that LMP1 was expressed in 78% NPC samples [10]. The region of LMP1 thought to be important for oncogenesis is the C-terminus which is a hot spot region for mutations [11]. Deletion of a 30-bp sequence in the LMP1 gene results in progression from a non-oncogenic to an oncogenic state. Restoration of the 30-bp sequence reversed the transformation ability [12]. The 30-bp deletion has been found in Hodgkin's disease (HD) [13], human immunodeficiency virus-related HD cases [14], Malaysian and Danish post-transplant lymphoproliferative diseases (PTLs) [13], nasal T/natural killer (NK)-cell lymphoma [15], Burkitt's lymphoma and non-Hodgkin's diseases [16]. The 30-bp deletion has also been shown to result in a more aggressive phenotype of EBV-associated lymphoproliferative disease and lymphoma *in vivo *[17].

Other mutations that have been identified in LMP1 gene include a point mutation at nucleotide position 169425 (G → T) resulting in loss of *Xho*I restriction site in exon 1, and multiple point mutations [18]. The *Xho*I polymorphism was also present in all NPC from Alaska and in some of the NPC samples from Caucasian Americans but was absent in the Africa's NPC and healthy controls [19,20]. These variations are represented in the well-studied EBV strains namely, CAO and C1510 [18,21]. It has been suggested that these changes in both CAO and C1510 are associated with increased tumorigenicity in SCID and nude mice [22]. The *Xho*I polymorphism has been found in EBV isolates in HD [23], nasal and peripheral T-cell lymphoma (NPTL) and infectious mononucleosis (IM) [24].

There are only a few reports on the association of these variants with clinicopathological data in NPC. The presence of the 30-bp deletion was strongly associated with non-keratinizing carcinoma type of NPC in one study [25]. However, no statistical significance in age, gender, radiosensitivity and pathological classification was reported in another study [26]. A study by Tan et al. [27], showed the coexistence of the wild type and 30-bp deletion in NPC biopsies but no correlation with clinicopathological data was made.

Hence, to date, the correlation between the co-existence of the LMP-1 30-bp deletion and loss of *Xho*I restriction site variants in either NPC tissue or plasma with clinicopathological data has not yet been studied. The objectives of our study was to determine the relationship between the LMP-1 variants with 30-bp deletion and/or loss of *Xho*I restriction site as well as the co-existence of these variants in NPC tissues and plasma with population characteristics and histological type.

## Methods

### Sample collection

A total of 42 NPC and 10 non-malignant nasopharyngeal formalin-fixed paraffin embedded tissue were used for this study. The non-malignant tissues were obtained from patients with suspected cases of NPC but were confirmed to be normal by histology. Sections of 4 μm thickness were cut from each tissue blocks. The specimens were histologically classified into three types according to World Health Organization (WHO) classification: Keratinizing squamous cell carcinoma (SCC, Type I), non-keratinizing carcinoma (NKC, Type II), and undifferentiated carcinoma (UC, Type III).

Blood samples were collected from another group of 35 patients with histopathologically confirmed NPC at Hospital Kuala Lumpur (HKL) from June 2006 to April 2007. Approximately 5 ml of peripheral blood was collected into a tube containing EDTA anticoagulant. EBV was recovered from the supernatant above the mononuclear cell layer after Ficoll-hypaque centrifugation at 2500 rpm for 20 minutes and frozen at -80°C until further processing. This supernatant was referred to as "plasma".

Ethics approval was obtained from the Ministry of Health Ethics Committee and Faculty of Medicine and Health Sciences, University Putra Malaysia Ethics Committee for this study.

### Cell culture

Cell line B95.8 was used as a positive control. The B95.8 cell line was grown in 37°C, 5% carbon dioxide (CO_2_), 95% humidifying air incubator and cultured with complete RPMI 1640 medium (GIBCO, USA) supplemented with 2mM L-glutamine, 100 IU/ml penicillin, 100 μg/ml streptomycin, 0.5 μg/ml Fungizone, 0.1 μg/ml Gentamycin (GIBCO, USA), 2.0 g/L sodium bicarbonate (Sigma, USA) and 10% foetal bovine serum (FBS) (GIBCO, USA). The cells were grown until 70% to 90% confluence before being harvested.

### DNA extraction

DNA from cell culture and paraffin-embedded tissue were extracted by using the GENE ALL™ Tissue SV (plus) mini kit (General Biosystem, Korea). An extra step of deparaffinization with xylene was performed for the tissues. Absolute ethanol was added to the cell pellet to remove the residual xylene and then air-dried. The tissue pellet was resuspended in 180 μl Tissue Lysis solution and digested overnight with 20 μl of 20 mg/ml Proteinase K at 56°C. Three μl of 20 mg/ml RNase A solution (Amresco, Ohio) was added, mixed thoroughly and incubated at room temperature to obtained an RNA-free DNA. Four hundred μl Tissue Binding Solution was added and the mixture was transferred to the spin column and centrifuged for 1 min at 10,000 rpm. Purification of DNA was performed according to manufacturer's operating instructions.

Plasma DNA was extracted using the GENE ALL™ Blood SV mini kit (General Biosystem, Korea). A total of 800 μl of plasma samples were used for DNA extraction per column. 80 μl of 20mg/ml Proteinase K solution (General Biosystem, Korea) and 3 μl of 20mg/ml RNase A (Amresco, Ohio) were added and incubated for 15 min at room temperature. Eight hundred μl of blood lysis buffer was added and mixed thoroughly by vortexing followed by 10 min incubation at 56°C. Eight hundred μl of absolute ethanol was added followed by pulse-vortex mixing. After centrifugation, the mixture was washed by following the protocols and solutions of the manufacturer. DNA was eluted by adding 30 to 60 μl of elution buffer (10mM TrisCl, pH 9.0, 0.5mM EDTA).

### Polymerase Chain Reaction (PCR)

For LMP1 30-bp deletion analysis, 2 μl of DNA, 1X PCR buffer, 1.5mM MgCl_2_, 200 nM dNTPs mix, 500 nM L30F (primer sequence 5'-GTCATAGTAGCTTAGCTGAAC-3') and L30R primers (primer sequence 5'-GAAGAGGTTGAAAACAAAGGA-3) [28] and 0.05U/μl Taq DNA polymerase (Fermentas, Canada) were used to carry out the PCR amplification. The PCR was performed by using T-Gradient Thermoblock (Biometra, Germany) with initial denaturation at 95°C for 5 min followed by 35 cycles of denaturation at 95°C for 1 min, annealing at 51.2°C for 1 min and extension at 72°C for 1 min. The final extension step was carried out at 72°C for 5 min. For *Xho*I restriction site analysis, the primers used were X1.1 (primer sequence 5'-ATGGAACACGACCTTGAGAGG-3') and X1.2 (primer sequence 5'-AACAGTAGCGCCAAGAGCAG-3') [13] and the annealing temperature was modified to 55.9°C. The PCR product for the *Xho*I polymorphism analysis was purified using the GENE ALL™ PCR SV kit (General Biosystem, Korea) following the manufacturer's recommendations.

### XhoI restriction enzyme digestion of PCR products

The purified PCR product was subjected to digestion with restriction enzyme *Xho*I (Fermentas, USA). Wild type B95.8 served as the positive control. The reaction mixture consisted of 1X buffer R, 0.05U/μl of restriction enzyme *Xho*I, 12 μl of purified PCR product and 5 μl of sterile distilled water was added to make up a total volume of 20 μl. The reaction was incubated at 37°C for 3 hours and subjected to heat inactivation at 50°C for 20 min.

### Agarose gel electrophoresis

The digested PCR product was analysed using gel electrophoresis with 3% low melt Agarose II gel (Amresco, Ohio). Electrophoresis was performed at 80 volts for 45 min in 1X TAE buffer. The gel was then stained with ethidium bromide and visualised by using FluorChem Imaging System (Alpha Innotech, USA). The Gene Ruler™ DNA ladder mix (Fermentas, USA) was used as the standard DNA molecular weight marker.

### DNA sequencing

To confirm that the amplicons harbour LMP-1 30-bp deletion, two amplicons from NPC tissue DNA were randomly selected for sequencing by using the BigDye^® ^Terminator v3.1 sequencing kit and ABI PRISM^® ^377 Genetic Analyser. Primers used for sequencing were L30F and L30R. The sequencing results were compared with the other EBV strains to determine the difference in the nucleotide sequences, if any.

### Statistical analysis

Fisher's exact test was used to analyse the association between the presence of the LMP1 30-bp deletion variant and *Xho*I polymorphism with population characteristics and histological data. Data were processed with the SPSS programme for Windows, version 13.0 (SPSS Inc., Chicago, Illinois).

## Results

### Detection of the EBV LMP1 gene 30-bp deletion

**Figure 1:**
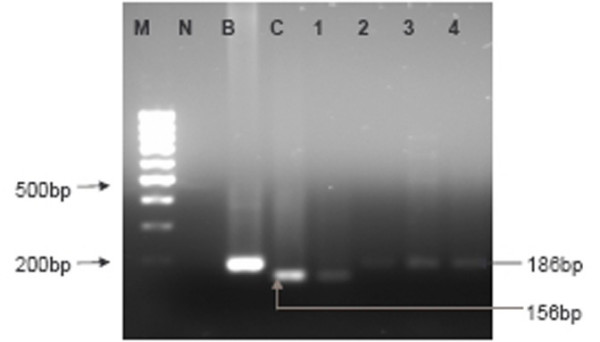
**Representative agarose gel electrophoresis of the LMP1 exon 3 amplicons with and without the 30-bp deletion**. M-100 bp DNA ladder marker, N-No template control, B-LMP1 exon 3 from B95.8, C-NPC tissue that display 30-bp deletion (product size= 156-bp), 1-NPC plasma that display 30-bp deletion (product size = 156-bp), 2–4-NPC plasma without the 30-bp deletion (product size = 186-bp).

The region spanning the LMP1 30-bp deletion was successfully amplified from 34/42 NPC cases and 8/10 non-malignant tissues. Among the 34 amplifiable cases, 47.1% (16/34) were from undifferentiated carcinoma (UC, WHO type III) samples, followed by 35.3% (12/34) in non-keratinizing carcinoma (NKC, WHO type II) and 17.6% (6/34) in keratinizing squamous cell carcinoma (SCC, WHO type I) samples. The LMP1 30-bp deletion variant (as represented in Lane C, Figure 1) was found in 19/34 cases (55.9%) of which 13 were undifferentiated carcinoma (type III) and 5 were non-keratinizing carcinoma (Type II) and 1 was keratinized squamous carcinoma (type I). The 30-bp deletion variant was not present in any of the 8 amplifiable non-malignant tissues (Table 1). A statistical different was found between the presence of LMP1 30-bp deletion in NPC versus non-malignant nasopharyngeal tissues (*p *= 0.005 Fisher's Exact test).

**Table 1:** 30-bp deletion and *Xho*I polymorphism in NPC biopsies and plasma, and non-malignant nasopharyngeal tissues

	NPC tissues % (n = 42)	Non-malignant nasopharyngeal tissues % (n = 10)	NPC plasma % (n = 35)
**Analysis**			
LMP1 30-bp deletion			
Frequency detected by PCR	81.0 (34/42)	80 (8/10)	82.9 (29/35)
With deletion	55.9 (19/34)	0	24.1 (7/29)
No deletion	44.1 (15/34)	100 (8/8)	75.9 (22/29)
With and without deletion	0 (0/34)	0 (0/8)	17.2 (5/29)
*Xho*I polymorphism			
Frequency detected by PCR	92.9 (39/42)	80 (8/10)	85.7 (30/35)
Loss of *Xho*I site	87.2 (34/39)	0	36.7 (11/30)
Retention of *Xho*I site	12.8 (5/39)	100 (8/8)	63.3 (19/30)
Co-existence of 30-bp deletion and *Xho*I-loss	59.4 (19/32)	0 (0/8)	25.0 (6/24)

**Figure 2:**
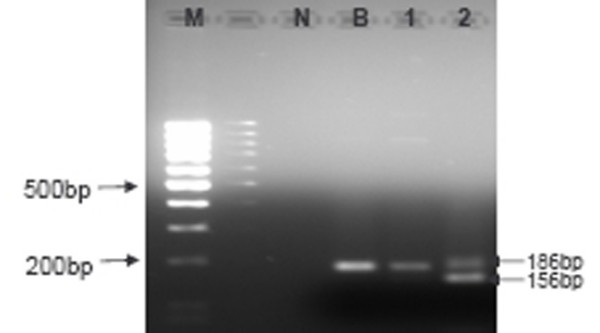
**Representative gel electrophoresis of LMP1 amplicons from NPC plasma**: M-100 bp DNA ladder marker, N-No template control, B-LMP1 exon 3 from B95.8, 1-NPC plasma without 30-bp deletion (product size = 186-bp), 2-NPC plasma with coexistence of wild type and 30bp deletion.

As for the plasma samples, 82.9% (29/35) of plasma samples were amplifiable for LMP1 30-bp deletion analysis. Seven out of 29 cases (24.1%) harboured the 30-bp deletion (as represented in Lane 1, Figure 1) whereas, 22/29 (75.9%) specimens retained the wild type variant (represented in Lane 2–4, Figure 1). Interestingly, of these 7 positive cases, concurrent expression of both variants with and without the LMP1 30-bp deletion were observed in 5 cases (5/29;17.2%) as represented in Lane 2, Figure 2 (Table 1).

The presence of LMP1 30-bp deletion was confirmed by sequencing two samples, namely NPC 1 and NPC 2 (Table 2). The samples which harboured the LMP1 30-bp deletion as determined by DNA sequencing were used as the controls in this study. Besides the 30-bp deletion, both NPC 1 and 2 showed the following substitutions: Q334R, L338S, and S366T between codon 327 and 383 of the carboxyl terminal of LMP1 as shown in Table 2. A point mutation from A → T at 168295 was also found in these two isolates. In addition, NPC 2 harboured one additional change at codon 335 (GGC → GAC) resulting in an amino acid change from glycine (G) to aspartic acid (D). This was also detected in the China 1 and DV strain.

**Table 2:** Comparison of deduced amino acid sequences in the LMP1 exon 3 in NPC1 and NPC2

**Nucleotide**	168342																		
**Codon**	**327**	**328**	**329**	**330**	**331**	**332**	**333**	**334**	**335**	**336**	**337**	**338**	**339**	**340**	**341**	**342**	**343**	**344**	**345**
B95.8	V	E	N	K	G	G	D	Q	G	P	P	L	M	T	D	G	G	G	G
CAO		A						R				S							
China I								R	D			S							
NPC 1								R				S							
NPC 2								R	D			S							

**Nucleotide**	168342																		

**Codon**	**346**	**347**	**348**	**349**	**350**	**351**	**352**	**353**	**354**	**355**	**356**	**357**	**358**	**359**	**360**	**361**	**362**	**363**	**364**

B95.8	H	S	H	D	S	G	H	G	G	G	D	P	H	L	P	T	L	L	L
CAO	*	*	*	*	*	*													
China I	*	*	*	*	*	*													
NPC 1	*	*	*	*	*	*													
NPC 2	*	*	*	*	*	*													

**Nucleotide**	168342																		

**Codon**	**365**	**366**	**367**	**368**	**369**	**370**	**371**	**372**	**373**	**374**	**375**	**376**	**377**	**378**	**379**	**380**	**381**	**382**	**383**

B95.8	G	S	S	G	S	G	G	D	D	D	D	P	H	G	P	V	Q	L	S
CAO		T																	
China I		T																	
NPC 1		T																	
NPC 2		T																	

Asterisks (*) indicate amino acid deletion. Previously published prototype sequences were obtained from Fennewald *et al*., 1984 (B95.8).

Hu *et al*., 1991 (CAO), Sung *et al*., 1998 (China 1). NPC 1 and NPC 2 are isolates from NPC tissue samples subjected to DNA sequencing.

Histological type and population characteristics including age, gender and race corresponding to the presence or absence of LMP1 30-bp deletion were compared. Statistical analysis showed that the presence of 30-bp deleted variant in NPC tissues between the keratinizing squamous cell carcinoma (SCC, WHO type I) and undifferentiated carcinoma (UC, WHO type III) was significantly different (*p *= 0.011; Table 3). Comparison of the races showed that 88.9% of Chinese (16/18) had the LMP1 30-bp deletion variant, followed by 20% Malay (3/15). The presence of LMP1 30-bp deletion from Chinese was statistically higher than Malay in NPC tissues (*p *< 0.05) (Table 3). No statistical difference was found with age and gender in NPC tissues (age, *p *= 0.289; gender, *p *= 1.000).

**Table 3:** Association between population characteristics and histological type with 30-bp deletion and *Xho*I-loss studiesin NPC tissue and plasma samples

	**Number of 30-bp deletion in LMP1 (%)**				**Number of Loss of *Xho *I site (%)**				**Co-existence of 30-bp deletion and *Xho *I-loss in NPC tissues**	
	
	NPC tissues	*P *value	NPC plasma	*P *value	NPC tissue	*P *value	NPC plasma	*P *value	Number of co-existence	*P *value
**Clinicopathological Features**										
**Sex**										
Male	14/25 (56.0)	1.000	7/26 (26.9)	1.000	24/27 (88.9)	0.615	9/24 (37.5)	0.529	14/23 (60.9)	0.689
Female	4/8 (50.0)^¶^		0/2 (0.0)^¥^		9/11 (81.8)^€^		0/2 (0.0)^Ω^		4/8 (50.0)^Ж^	
**Age**										
<50 years	6/14 (42.9)	0.289	4/15 (26.7)	1.000	12/15 (80.0)	0.630	4/16 (25.0)	0.234	6/13 (46.2)	0.274
>50 years	11/17 (64.7)^§^		2/11 (18.2)^∞^		19/21 (90.5)^∂^		5/10 (50.0)^Ω^		11/16 (68.8)Φ	
**Race**										
Malay	3/15 (20.0)	Malay vs. India, *p *= 1.000 Malay vs. Chinese, *p *< 0.05* Chinese vs. India, *p *= 0.158	1/10 (10.0)	0.363	14/19 (73.7)	Malay vs. Chinese, *p *= 0.046*	3/11 (27.3)	0.689	3/14 (21.4)	Malay vs. India, *p *= 1.000
Chinese	16/18 (88.9)		5/17 (29.4)Ø		19/19 (100.0)		7/17 (41.2)^Ψ^		16/17 (94.1)	Malay vs. Chinese, *p *< 0.050*
Indian	0/1 (0.0)				1/1 (100.0)				0/1 (0.0)	
**Histological type**										
Type I – Keratinizing squamous cell carcinoma (SCC)	1/6 (16.7)	SCC vs. NKC, *p *= 0.600	0/2 (0.0)	SCC vs. NKC, *p *= 1.000	2/5 (40.0)	SCC vs. NKC, *p *= 0.063	0/2 (0.0)	SCC vs. NKC, *p *= 0.429	1/4 (25.0)	SCC vs. NKC, *p *= 1.000
Type II – Non-keratinizing carcinoma (NKC)	5/12 (41.7)	SCC vs. UC, *p *= 0.011*	2/6 (33.3)	SCC vs. UC, *p *= 1.000	14/16 (87.5)	SCC vs. UC, *p *= 0.006*	4/6 (66.7)	SCC vs. UC, *P *= 1.000	5/12 (41.7)	SCC vs. UC, *p *= 0.061
Type III – Undifferentiated carcinoma (UC)	13/16 (81.3)	NKC vs. UC, *p *= 0.050	1/8 (12.5)^¤^	NKC vs. UC, *p *= 0.538	18/18 (100.0)	NKC vs. UC, *p *= 0.214	1/10 (10.0)^θ^	NKC vs. UC, *p *= 0.056	13/16 (81.3)	NKC vs. UC, *p *= 0.050

Statistical significance of the difference was analyzed using Fisher's Exact test, (*) indicates statistical significance (p < 0.05). ^¶^Data was only available for 33 out of 34 cases.^§^Data was only available for 31 out of 34 cases. ^¥^Data was only available for 28 out of 29 cases. ^∞^Data was only available for 26 out of 29 cases. ^Ø^Data was only available for 27 out of 29 cases. ^¤^Data are only available for 16 out of 29 cases. ^€^Data was only available for 38 out of 39 cases. ^∂^The data was only available for 36 out of 39 cases. ^Ω^Data was only available for 26 out of 30 cases. ^Ψ^Data was only available for 28 out of 30 cases. ^θ^Data was only available for 18 out of 30 cases for NPC plasma samples. ^Ж^Data was only available for 31 out of 32 cases for NPC tissues. ^Φ^Data was only available for 29 out of 32 cases for NPC tissues.

### Detection of the XhoI polymorphism in the exon 1 of LMP1 gene

**Figure 3:**
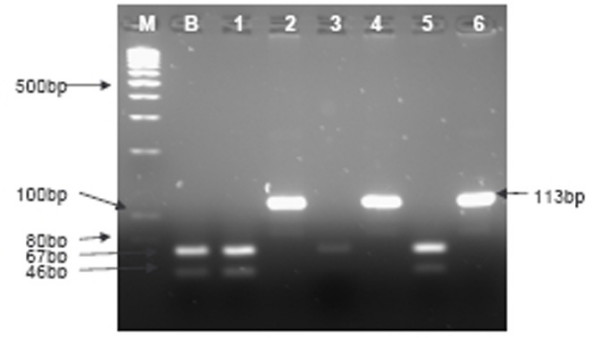
***Xho *I restriction digest analyses of NPC and non-malignant tissues, and plasma samples from NPC patients**. M-100-bp DNA ladder marker, B-B95.8 control that with *Xho*I restriction site (two bands of 67 and 46-bp after *Xho*I digestion), 1-NPC tissue sample with *Xho*I restriction site, 2-NPC tissue sample with loss of *Xho*I site show undigested 113-bp product, 3-Non-malignant nasopharyngeal tissue that retained *Xho*I restriction site, 4&6-NPC plasma samples with loss of *Xho*I site show undigested 113-bp product, 5-NPC plasma samples with *Xho*I restriction site.

As summarized in Table 1, *Xho*I polymorphic region was successfully amplified in 92.9% (39/42) NPC and 80% (8/10) of non-malignant tissues. PCR with primers X1.1 and X1.2 generates a 113-bp amplicon which harbours the *Xho*I polymorphic region. If *Xho*I site is present, restriction enzyme digest of the 113-bp amplicon yields two DNA fragments of 67 and 46-bp. The loss of *Xho*I restriction site was detected in 87.2% (34/39) of NPC tissues samples (represented in lane 2, Figure 3). All 8 of the amplifiable non-malignant tissues samples retained the *Xho*I restriction site (represented in lane 3, Figure 3). The loss of *Xho*I restriction site was statistically higher in the NPC tissues compared to the non-malignant tissues (*p *< 0.05; Fisher's Exact test). As for the plasma sample, the region spanning the *Xho*I restriction site in exon 1 was successfully amplified in 85.7% (30/35) of the samples. The loss of *Xho*I site was found in 11/30 cases (36.7%), (represented in lane 5, Figure 3).

The presence of EBV variant with loss of *Xho*I restriction site with population characteristics and histological type are as summarised in Table 3. Statistical difference was found only for the histological type and race. In this study, the loss of *Xho*I restriction site was detected in 40.0% (2/5) of the keratinizing squamous cell carcinoma (SCC, WHO type I) samples, 87.5% (14/16) of the non-keratinizing carcinoma (NKC, WHO type II) samples, and 100.0% (18/18) of the undifferentiated carcinoma (UC, WHO type III) samples (Table 3). Statistical analysis showed a significant difference for the presence of *Xho*I polymorphism in NPC tissues between the SCC type and UC type (*p *= 0.006; Table 3). Loss of *Xho*I restriction site was found in 100.0% of Chinese (19/19) and Indian (1/1), followed by 73.7% Malay (14/19). The presence of EBV variant with *Xho*I-loss in NPC tissues from Chinese was statistically higher than Malay (*p *= 0.046).

### Co-existence of the LMP1 30-bp deletion and XhoI-loss in NPC

Table 1 shows 19/32 NPC tissues and 6/24 plasma samples harboured both LMP1 30-bp deletion and *Xho*I loss. Table 3 shows the association between the coexistence of the LMP1 30-bp deletion and loss of *Xho*I in 19/32 (59.4%) of NPC tissues. Statistical significance was only found in NPC tissues between Chinese and Malays. No significant relationship was found between co-expression of both variants and histological type in NPC.

## Discussion

In this study, the presence of a high frequency of the LMP1 30-bp deletion in EBV isolates from NPC tissues as compared to non-malignant tissues is in agreement with previous reports [21,27,28]. Interestingly, although the 30-bp deleted variant was not detected in non-malignant tissues, it has been found in lymphoblastoid cell lines derived from Chinese healthy chronic carriers [3]. The authors suggested that the presence of variant LMPl in NPC simply reflects the overall prevalence of this polymorphism in EBV strains infecting the general Chinese population.

The incidence rate of the 30-bp deletion variant in this study is lower than that compared to three other reports from Malaysia, whereby, positive rates of 97%, 91% and 100% were reported [27,29,30]. Other studies from NPC endemic areas such as Southern China and Taiwan showed 75% and more than 90% deleted LMP1-positive cases, respectively [18,31-33]. The reasons for the lower incidence rate in this study is unclear.

Another difference in our data is that coexistence of dual variants with and without 30-bp deletion in plasma but not NPC tissues from another group of patients. This differs from other studies, whereby, existence of dual variants in 1.5% (8/542) and 16% (4/25) NPC tissues have been previously reported [26,27]. To the best of our knowledge, this is the first report to show the co-presence of both variants in plasma from NPC patients. It is most likely that EBV in plasma is derived from the tumour as shown by Chan *et al*. (2003) [34]. We are not able to confirm this in the current study as we have not determined the LMP1 30-bp status with paired tissue samples.

In our study, the DNA sequence of two of the amplicons from NPC tissues spanning the LMP1 C-terminal region, namely NPC1 and NPC2 was determined. In addition to the 30-bp deletion, NPC2 but not NPC1 harboured a mutation at codon 335. This sequence resembles the China 1 [31] and DV-Asp335 strain [32]. The codon 335, which is located outside the known functional domains (CTAR1 and CTAR2) is involved in the nuclear factor (NF)-κβ signaling and has been postulated to be involved in protein turnover [35]. Comparison of the LMP1 variants isolated from healthy individuals in Hong Kong showed that 80% of these individuals harboured the 30-bp deletion but the majority of them (73%) do not have the substituted amino acid at codon 335. The prevalence of codon 335 mutation in our NPC patients in Malaysia has not yet been determined.

Similar to the LMP1 C-terminus, a number of sequence variations have been found in the short cytoplasmic N-terminus. Among them is the loss of an *Xho*I restriction site in exon 1 which is commonly reported in Southern China. This was first described in the CAO strain, the nude mouse passaged Chinese NPC EBV isolate. The presence of *Xho-*I loss variant in our tissues samples is comparable to other endemic areas such as in Southern China and Taiwan, whereby, 97–100% of NPC cases harboured loss of *Xho*I restriction site [20,36]. This polymorphism was also present in NPC from Alaska and in some of the NPC samples from Caucasian Americans but was absent in NPC and healthy controls in Africa [19,37]. The loss of an *Xho*I restriction site has been associated with Chinese NPC, and it has been considered to be a specific tumour marker in NPC biopsies and throat washes [18,21,27,38]. However, to date, little is known about the efficiency of malignant transformation by this variant.

In this study, NPC tissues showed a higher percentage of *Xho*I-loss as compared to plasma samples from another group of patients. The high incidence of *Xho*I-loss is comparable to another study, whereby, an incidence rate of 93% (25/27) of NPC biopsies was reported [27]. No reports on *Xho*I-loss variants have been reported for plasma samples.

In order to further understand the role of LMP1 variants in NPC pathogenesis, we performed statistical analysis between either the presence of 30-bp deleted LMP1 or *Xho*I-loss with histological type and population characteristics such as gender, age and race. Our data suggested that EBV-associated NPC mutations in LMP1 occurred at different rates in different racial groups. This is consistent with another study, whereby, the LMP1 30-bp deletion variant was found to be predominant in Inuit origin populations [39] and Chinese [21] which were the two groups who are especially prone to NPC [1].

Our study showed that EBV LMP1 30-bp deletion and *Xho*I-loss variants were found to be predominant in undifferentiated carcinomas (Type III) compared to keratinizing squamous cell carcinoma (Type I). This suggests that the variants may play a crucial role in carcinogenesis of undifferentiated carcinomas (Type III). No correlation with *Xho*I-loss and histological type has been reported. Comparison with published data showed that there is no relationship between histological type and LMP1 30-bp deletion variant in NPC [26] which differs from our study. EBV isolates with the LMPl 30-bp deletion have been preferentially found in histologically aggressive forms of Hodgkin's disease [40,41]. However, findings by Khanim *et al*. (1996) [37] do not support the association of LMP1-deleted EBV with aggressive HD. The possibility that LMP1 deletions may contribute to the malignant behaviour of NPC cases constitutes an attractive hypothesis that deserves further investigation.

Interestingly, majority of our NPC tissues (19/32; 59.4) showed the coexistence of both the 30-bp deletion and the loss of *Xho*I restriction site. This resembles the CAO, C1510, China 1 and DV2 isolates from NPC endemic areas. The clinical significance of coexistence of both variants is unknown. The 30-bp deletion alone is not a prognosticator for overall survival [26,28] and distant metastasis [26]. Further investigation is required to determine if coexistence of the two variants is correlated with worse prognosis and overall survival of our patients. We also found a higher incidence rate of coexistence of 30-bp deletion and *Xho*I loss variants in Chinese versus Malay patients. However, there was no relationship between coexistence of these variants with histological type. The implications of these findings are unclear and require further investigation.

## Conclusion

In conclusion, LMP1 30-bp deletion and loss of *Xho*I site was found in NPC tissues but not non-malignant tissues. Dual variants of LMP1 were only found in plasma from NPC patients. A significant relationship was found between LMP1 30-bp deletion and loss of *Xho*I site between histological type and race. The prevalence of certain EBV strains as represented by LMP1 sequence variation in NPC, particularly the 30-bp deletion in C-terminus and *Xho*I polymorphism in N-terminus in LMP1 as shown in this study as well as previous studies, may have unique functional properties, which determine disease association or development. Further studies with these variants are needed to elucidate the LMP1 signalling pathway, and to assess the contribution of LMP1 sequence variation to the pathogenesis of EBV-associated tumours, particularly in NPC.

## Abbreviations

Nasopharyngeal carcinoma; Epstein-Barr virus (EBV), latent membrane protein 1 (LMP1), 30-bp deletion, *Xho*I loss.

## Competing interests

The author(s) declare that they have no competing interests.

## Authors' contributions

HFSand HSSplanned the experiment and performed most of the experiments with the help and supervision of HFS. WKYselected the blocks, performed the paraffin tissues sectioning and prepared patient data. YYY provided the plasma samples and applied for ethics approval. HSSand HFSwere responsible for data analysis and preparation of the manuscript. All authors read and approved final version of the manuscript.
